# The research progress on synchronous endometrial and ovarian carcinoma

**DOI:** 10.3389/fonc.2023.1291602

**Published:** 2023-12-06

**Authors:** Wenli Gan, Ce Bian

**Affiliations:** ^1^ Department of Gynecology and Obstetrics, Key Laboratory of Birth Defects and Related Diseases of Women and Children, Ministry of Education, West China Second Hospital, Sichuan University, Chengdu, Sichuan, China; ^2^ Department of Gynecology and Obstetrics, Affiliated Hospital of Sichuan Nursing Vocational College (The Third People's Hospital of Sichuan Province), Chengdu, Sichuan, China

**Keywords:** endometrial neoplasia, ovarian neoplasia, synchronous, risk factor, prognosis

## Abstract

Synchronous endometrial and ovarian carcinoma (SEOC) is the most common combination of primary double cancer in the female reproductive system. The etiology and pathogenesis of SEOC remain unclear, and clinically, it is often misdiagnosed as metastatic cancer, affecting the formulation of treatment plans and prognosis for patients. This article provides a review of its epidemiology, pathological and clinical characteristics, risk factors, pathogenesis, diagnosis, treatment, and prognosis.

## 1 Introduction

In the female reproductive system, primary double cancer is relatively rare, accounting for approximately 0.63-1.7% of all malignant tumors in the female reproductive system. Among them, synchronous endometrial and ovarian carcinoma (SEOC) is the most common, accounting for about 40-51.7% (1–3). Primary double cancer is often misdiagnosed as metastatic cancer in clinical practice, making it challenging for both clinicians and pathologists to accurately differentiate between primary and metastatic cases. This differentiation is of crucial importance for clinical management, treatment decisions, and patient prognosis. This article aims to provide a comprehensive review of the clinical and pathological characteristics, types, diagnosis, treatment, and prognosis of SEOC, with the goal of offering guidance for clinical diagnosis, differentiation, and personalized treatment.

## 2 Epidemiology

Synchronous Endometrial and Ovarian Carcinoma (SEOC) is a malignant tumor that occurs simultaneously in the endometrium and ovaries, and it is the most common type of primary double cancer in the female reproductive system. However, previous research reports have shown significant variations in its incidence, which can be attributed to factors such as ethnicity, geographical location, and small sample sizes. For example, Eisner et al. (1) studied 3,863 female reproductive tract cancer patients registered at the University of California, Los Angeles, between 1955 and 1986, and found 26 cases (0.7%) of primary double cancer, including 11 cases of SEOC (0.3%). In another study conducted in Turkey, SEOC accounted for approximately 0.89% (2). Previous research has reported SEOC incidence rates of less than 3% in ovarian cancer patients (4–7) and approximately 3.0-5.5% in endometrial cancer patients (5, 8–12).

## 3 Pathological characteristics

In SEOC, the histological types of tumors in both sites may be the same or different, but the most common type in both sites is endometrioid carcinoma. Other types, such as mucinous, clear cell, and mixed-type tumors, can also occur. It has been reported that endometrioid carcinoma in both sites accounts for about 45.5-86% of cases (11, 13–15). In the study of Zaino et al. (13), 74 patients with SEOC were included, almost 90% (67 patients) of tumors identified in the ovary and 88% (65 patients) of tumors identified in the endometrium were of endometrioid cell type (with or without foci of squamous differentiation), and the proportion of patients with endometrioid histological types at both sites was as high as 86%(64 patients), and most tumors were well differentiated. In 51% (38 patients) of these cases, both endometrial and ovarian tumors were histologically grade 1. In contrast, among epithelial ovarian cancers, endometrioid ovarian carcinoma represents only about 11% of cases (16).

## 4 The pathological diagnosis and differential diagnosis

In 1985, Ulbright and Roth (17) first attempted to use pathological features to differentiate between metastatic cancer and independent double primary cancers. The diagnosis of metastatic cancer was primarily based on the criteria of multiple ovarian nodules, with the following as secondary criteria: small ovaries (< 5 cm), involvement of both ovaries, deep myometrial invasion, vascular invasion, and involvement of the fallopian tube lumen. In 1998, Scully (18) modified and proposed the following clinical-pathological diagnostic criteria based on these criteria: 1, Histological differences between tumors.2, Endometrial tumors with no or only superficial myometrial invasion.3, Endometrial tumors without invasion of vascular spaces.4, No evidence of atypical endometrial hyperplasia.5, Absence of evidence of spread of other endometrial tumors.6, Unilateral ovarian tumor (80-90%). 7, Ovarian tumor located in the parenchyma.8, No invasion of vascular spaces, surface implantation, or primary location at the ovarian hilum.9, Absence of evidence of spread of other ovarian tumors.10, Ovarian endometriosis.11, Different ploidy or DNA index in the tumor (if it’s non-diploid). 12, Different molecular genetic or cytogenetic abnormalities in the tumor. Many retrospective studies have confirmed the favorable survival outcomes of patients diagnosed using the Scully criteria for independent primary double cancer. This has led to the continued clinical and pathological use of these criteria to date. In 2018, Yang et al. (19) in a study conducted by the Affiliated Cancer Hospital of Tianjin Medical University in China, refined the Scully criteria and added clinical staging, foming 8 criteria (unilateral/bilateral ovarian involvement, ovarian tumor size, endometrial myometrium infiltration depth, lymphovascular infiltration status, involvement of other sites, endometriosis of ovary, atypical endometrial hyperplasia, and tumor staging), and gave each standard a value, making it a standard and regrouping 52 patients with SEOC. Then, they found that the new criteria can better distinguish metastatic cancer from primary double cancer compared with Scully standard. However, due to the small sample size and no control group in the study, this scoring standard was not widely used.

Previous studies have used some molecular detection techniques, including X chromosome inactivation pattern, gene mutation detection (P53, K-Ras, PTEN, PIK 3CA, POLE and CTNNB 1), immunohistochemistry, vimentin expression, loss of heterozygosity, microsatellite instability, etc.to assist in the diagnosis and differentiation of SEOC (20–25). The study conducted by Dirk Brinkmann et al. (26) in 2004, involving 62 SEOC patients, revealed that the genetic analysis of allelic loss and microsatellite instability demonstrated a consistency of only 53% with histological diagnosis. This highlights the discordance between genetic and histopathological diagnoses and underscores the limitations of histological diagnosis in SEOC cases. In 2008, Ramus, S. J. et al. (25) conducted a study involving 90 SEOC patients, where a combination of histological and genetic analysis was used. Out of the 88 patients with clear diagnoses, genetic analysis was able to diagnose 64 patients who might have been missed by relying solely on histology. This demonstrates the effectiveness of genetic analysis in distinguishing between primary double cancer and metastatic cancer cases. In recent years, with the rapid advancement of second-generation gene sequencing technology, sequencing analyses based on this technology have proposed a molecular clonality association in the vast majority of SEOC cases, indicating that most SEOCs are the result of primary tumors accompanied by metastasis. These molecular clonality studies help to gain a deeper understanding of the pathological mechanisms and origins of SEOC (27–29). In a study conducted by Michael S. Anglesio et al. (27), 18 SEOC cases were analyzed to investigate the relationship between endometrial and ovarian components. Utilizing targeted sequencing and whole exome sequencing, 17 cases showed clonal relationships, indicating primary tumors with metastasis. This included 10 out of 11 cases that were classified according to clinical pathological standards as primary double cancers. In another study, 23 cases of SEOC were collected and analyzed, all of which had endometrioid carcinoma as pathological type, 15 of which were classified as independent primary tumors by clinicopathology, 5/23 were analyzed by whole exome sequencing, and the remaining 18 were analyzed by large-scale parallel sequencing.Targeting 341 (n=4) or 410 (n=14) key cancer genes, the results showed that 22 sporadic SEOC were associated with cloning (28). On this basis, the concept of “microenvironment confinement” of metastasis has been proposed in SEOC. This means that tumor cells in SEOC have the capability to detach from the primary lesion without undergoing apoptosis, spread spatially, and only re-locate within exclusive microenvironments without extensive metastasis. This distinguishes SEOC from endometrial or ovarian cancer, which often metastasize widely through lymphatic, hematogenous, or implantation routes. This phenomenon is also associated with the favorable prognosis observed in SEOC cases (27). While the studies mentioned above have provided evidence of clonality in SEOC, it’s important to note that due to the limitations of markers and detection methods, they may not comprehensively assess or qualify the clonality. Additionally, these studies may not definitively determine the clone’s origin and the direction of tumor metastasis, considering the potential heterogeneity within tumors. While the direction of metastasis is not definitively established, it is most likely that it occurs from the endometrium to the ovaries (30–32). Some research also suggests that the ovaries may be the preferred site of metastasis for tumors originating from various body parts (33). In a multicenter retrospective study conducted by Iacobelli, V. et al. (31) in 2020, it was found that the molecular characteristics of SEOC patients exhibited a remarkable similarity to the molecular profile of the uterine endometrial carcinoma tumor set from The Cancer Genome Atlas (TCGA) in 2013. This suggests that the endometrium might be the primary source of these cases rather than the ovaries. In clinical practice, when endometrial cancer spreads to the ovaries, it often presents as the invasion of the ovarian surface by multiple small tumor nodules and infiltration of vascular spaces.In contrast, primary endometrial tumors typically exhibit invasion into the deep layers of the uterine muscle and often extend into the fallopian tubes (34). Indeed, the clinical presentation of endometrial cancer spreading to the ovaries, as described, can be contradictory to Scully’s diagnostic criteria for distinguishing between primary double cancer and metastatic cancer. This highlights the complexity and challenges in accurately diagnosing and differentiating SEOC cases, as various factors and criteria may need to be considered for a comprehensive assessment. While the research mentioned points toward an endometrial origin for SEOC, it’s important to note that further extensive clinical studies are needed to provide definitive insights. This clarification is crucial for guiding surgical and subsequent adjuvant treatments to improve the prognosis and survival outcomes of SEOC patients.

## 5 Pathogenesis

As of now, the exact etiology and pathogenesis of SEOC remain unclear. Some researchers have proposed that the higher occurrence rate of SEOC compared to isolated endometrial or ovarian cancer may be due to the shared presence of several common risk factors, such as infertility and low parity (35). SEOC is more common in young, infertile and premenopausal women, indicating that the role of estrogen in the occurrence and development of SEOC is worthy of further investigation (14). Furthermore, the theory of embryonic origin supports the occurrence of SEOC (36–38). It suggests that the epithelial tissues of the cervix, uterus, fallopian tubes, and ovaries all originate from the Müllerian duct system. When these tissues are exposed to the same carcinogenic factors, it can lead to the independent development of multiple primary tumors. This further emphasizes the possibility that SEOC may involve the independent development of multiple primary tumors. Although endometrioid ovarian carcinoma represents a minority within epithelial ovarian cancers, it can account for up to 88% in SEOC (13). While most ovarian cancers occur at the fimbrial end of the fallopian tube, this location doesn’t explain all ovarian tumors, and some of these tumors likely originate from components of the secondary Müllerian duct system (36).

## 6 Risk factors

### 6.1 Age

Previous research has shown that SEOC is common among young, premenopausal women (12). In the study by Soliman, P. T. et al. (14). The median age at SEOC diagnosis was 50 years, with over 50% of cases occurring in premenopausal women. This is about 10 years younger than the median age of onset for single endometrial or ovarian cancer (37). Similar findings were observed in the research by Kobayashi, Y. et al. (39). These results suggest a potential association between SEOC incidence and female hormonal factors.

### 6.2 Infertility and parity

Research reports indicate that over 50% of SEOC patients suffer from infertility (39). In another retrospective study involving SEOC patients under the age of 40, the proportion of infertility patients reached as high as 81% (9). Infertility and low parity are recognized risk factors for both endometrial and ovarian cancers (40). In the study by Herrinton, L. J. et al. (35), SEOC was more likely to occur in infertility and low parity. However, the precise relationship and underlying mechanisms require further research for a comprehensive understanding.

### 6.3 Obesity

Previous studies have found that SEOC patients are more common in obese women (14, 41). Obesity is a risk factor for the development of endometrial cancer (42). This is due to the excessive peripheral conversion of androgens to estrogens in adipose tissue, leading to a high estrogen state in the body, which can increase the risk of developing endometrial cancer. It has also been found that women who are overweight or obese during adolescence or young adulthood have an increased risk of ovarian cancer compared to women with a moderate body mass index (18.5-24.9 kg/m2) (43). More studies are needed to determine whether obesity is related to the occurrence of SEOC.

### 6.4 Genetic factors

It is well known that some cases of endometrial cancer or ovarian cancer are associated with Lynch syndrome, also known as hereditary non-polyposis colorectal cancer syndrome (HNPCC). Lynch syndrome is an autosomal dominant genetic disorder caused by mutations in mismatch repair (MMR) genes. Women who carry MMR gene mutations have an increased risk of developing gynecological cancers, with a 43% risk of developing endometrial cancer and a 12% risk of developing ovarian cancer. This risk is more common in premenopausal women, with the peak incidence occurring between the ages of 45 and 55 (44). In patients with HNPCC, the occurrence of multiple primary cancers is not uncommon. In the clinical management and assessment of SEOC patients, consideration should also be given to their genetic susceptibility, especially in young premenopausal women. In the study conducted by Soliman PT. et al. (45), using IHC analysis and MSI testing in SEOC patients, it was found that 7% of patients had clinical or molecular criteria suggestive of Lynch syndrome. All of these patients had a history of HNPCC or a first-degree relative with the disease. In another study involving 32 SEOC patients with characteristic analysis of MMR proteins, the results suggested that most SEOC cases are not caused by hereditary cancer due to germline mutations (39). Although these research findings suggest that only a minority of SEOC patients have a history of HNPCC and a family history related to genetics, MMR gene mutation testing holds significant importance in clinical decision-making for SEOC patients who are young and wish to preserve their fertility. In the future, more large-scale prospective studies are needed to confirm its impact on patient treatment and prognosis.

## 7 Clinical diagnosis

Synchronous endometrial and ovarian carcinoma (SEOC) is often challenging to clinically diagnose. It can be mistaken for metastatic cancer, impacting treatment decisions and prognosis. Diagnosis typically involves a combination of clinical evaluation, imaging studies, and pathological examination of tissue samples from both the endometrium and ovaries. An accurate diagnosis is crucial for guiding treatment and improving patient outcomes.

### 7.1 Clinical manifestations and signs

SEOC patients lack specific clinical symptoms and signs. Compared to early-stage primary ovarian cancer, which is often asymptomatic, most SEOC cases benefit from the typical symptoms of endometrial cancer in the early stages, namely abnormal vaginal bleeding. Approximately 90% of endometrial cancer patients experience symptoms of abnormal vaginal bleeding, sometimes accompanied by uterine pus accumulation. The most common symptom in SEOC patients, as indicated by the majority of research findings, is abnormal vaginal bleeding, followed by abdominal pain, abdominal distension, and pelvic abdominal masses (5, 14, 39, 41). In advanced stages, SEOC can also manifest as ascites, cachexia, and compressive symptoms. These symptoms and signs are not necessarily specific indicators of SEOC, as they can also be related to other gynecological issues.

### 7.2 Serological markers

The value of CA125 in preoperative detection of endometrial or ovarian cancer is limited. In most cases of early-stage ovarian cancer or endometrial cancer, CA125 levels may not be elevated. It is primarily used clinically for disease monitoring and treatment assessment (46). In a study by Jain, V. et al. (41), a retrospective analysis of preoperative CA125 levels in SEOC patients showed that approximately 80% of patients had elevated CA125 levels with a median level of 150 IU/ml. Another study retrospectively analyzed 347 patients with epithelial ovarian cancer involving the uterus and found that about 82.8% of patients had CA125 levels greater than 100 IU/ml (47). This suggests that the specificity and sensitivity of CA125 as a preoperative diagnostic tool in SEOC patients need further research.

### 7.3 Imaging examination

Imaging examinations can play a significant role in the clinical diagnosis of SEOC. Commonly used imaging tests include ultrasound, computed tomography (CT), and magnetic resonance imaging (MRI).

#### 7.3.1 Ultrasound examination

Transvaginal ultrasound is often more sensitive and helps identify abnormalities in the ovaries and endometrium. Color Doppler ultrasound can provide information about tumor blood supply. In a retrospective observational study from Italy in 2019, the ultrasound characteristics of SEOC patients were compared to those of patients with ovarian metastases from endometrial cancer. The ovarian masses in SEOC patients showed unilateral multilocular or solid masses, while the ovarian masses in the metastatic group were mostly bilateral solid masses. Endometrial lesions in the synchronous group presented more often with no myometrial infiltration and less often with a multiple-vessel pattern on color Doppler compared with the endometrial lesions in the metastasis group. These differences in morphological features may aid in preoperative identification of the two types of cancer (48).

#### 7.3.2 Computed tomography (CT)

CT scans helps evaluate lymph node enlargement and the extent of spread within the abdominal cavity. For advanced-stage endometrial cancer, It can provide information about extrapelvic metastasis (46).

#### 7.3.3 Magnetic resonance imaging (MRI)

MRI can be used for assessing the depth of myometrial invasion, involvement of the cervical stroma, and lymph node metastasis in endometrial cancer cases (46).

## 8 Treatment

Due to the low incidence of SEOC and the difficulty in establishing a preoperative diagnosis, along with the limitations of retrospective studies with small sample sizes, there is currently no unified standard or consensus for the treatment of SEOC. However, some studies suggest that for early-stage and low-grade SEOC patients, surgical treatment alone may lead to favorable outcomes (5, 49, 50). While there is no standardized treatment approach at present, these research findings provide valuable insights into SEOC treatment. Clinicians need to tailor individualized treatment strategies based on each patient’s specific circumstances and pathological diagnosis. Furthermore, as further research and clinical practice progress, we may gain a better understanding of how to effectively manage SEOC. Clinical physicians can develop specific surgical plans based on the surgical standards for endometrial cancer or ovarian cancer, as well as the individual circumstances of the patient. After surgery, doctors will assess the risk factors to determine whether adjuvant treatment is necessary and develop specific adjuvant radiotherapy or chemotherapy plans as needed. This personalized treatment strategy can better meet the needs of patients, improve treatment effectiveness, and prognosis.

### 8.1 Surgical treatment

The preferred treatment for SEOC is surgery. The standard surgical approach involves staging surgery, which includes total hysterectomy, bilateral salpingo-oophorectomy, pelvic and para-aortic lymph node dissection, and omentectomy (5, 38). In a multi-center retrospective study from Turkey, which analyzed 63 cases of SEOC, it was proposed that the initial surgical standard for SEOC is optimal cytoreduction surgery (51). Some previous studies have suggested that lymph node dissection can improve the survival prognosis of SEOC patients through retrospective analysis, and they recommend lymph node dissection for all SEOC patients (52, 53). In primary endometrial or ovarian cancer, whether systemic lymphadenectomy improves survival is controversial (16, 46, 54).

Since SEOC is difficult to diagnose preoperatively, there may be cases where the surgical scope is insufficient. To date, there is no specific research to guide whether additional surgery or adjuvant treatment is needed after a clear pathological diagnosis following surgery. However, it may be determined based on the histological type and grading of the tissue, similar to the approach for single uterine or ovarian cancer. For example, if a surgery is performed preoperatively assuming endometrial cancer, postoperative comprehensive staging surgery and maximal cytoreductive surgery should be conducted, taking into account the histological subtypes of ovarian cancer. Afterward, adjuvant chemotherapy and maintenance treatment can be further determined (55).

### 8.2 Fertility preservation therapy

At present, fertility preservation therapy has been widely carried out in early stage and low-grade endometrial cancer or ovarian cancer for young women with fertility requirements, but there are few studies on fertility preservation therapy in patients with SEOC. In 2005, Morice, P. et al. (56) proposed that for patients with early stage and low-grade endometrial cancer who wanted conservative treatment, laparoscopic surgery should be performed to explore the adnexal and pelvic conditions to rule out extrauterine disease. Conversely, a multicenter retrospective study from Korean found that only 21 (4.5%) of 471 patients under 40 years of age with early endometrial cancer concurrent ovarian malignancy, and no synchronous cancer was found in low-risk endometrial cancer, so they do not advocate diagnostic laparoscopy in patients with early stage endometrial cancer who wish to be treated conservatively (9). In addition, study has also been reported two young patients with endometrioid borderline ovarian cancer whose final histopathological examination confirmed the diagnosis of invasive uterine cancer, suggesting that curettage is an essential way to rule out primary endometrial cancer before planned fertility-sparing surgery (57). The author believes that fertility preservation treatment for young SEOC patients needs multidisciplinary comprehensive evaluation, such as assisted reproductive technology, obstetrics, genetic counseling, etc., and the risk of subsequent disease progression and follow-up requirements of fertility preservation function should be fully informed. Existing studies have not mentioned such issues much, and more relevant studies are needed to guide clinical practice in the future.

### 8.3 Adjuvant therapy

Whether adjuvant therapy is necessary after surgery for SEOC is still a subject of debate. According to the study by Yoneoka et al. (58), SEOC patients with lesions limited to the uterine body and adnexa have a lower risk of recurrence and may not require adjuvant treatment. For patients with advanced stage, high-grade, poorly differentiated SEOC, and residual tumor tissue, aggressive adjuvant therapy is recommended (5, 53). Specific adjuvant treatment regimens can be tailored based on the adjuvant treatment methods used for endometrial cancer or ovarian cancer. For high-intermediate risk (HIR) subgroups in endometrial cancer, postoperative adjuvant radiotherapy is recommended as it can significantly reduce the risk of recurrence (46, 59). The HIR subgroup is defined by the Gynecologic Oncology Group (GOG) and the Post-Operative Radiation Therapy in Endometrial Cancer (PORTEC) for endometrial cancer. The definition of HIR in the PORTEC criteria includes the following two criteria: age greater than 60 years, grade 3 disease, or ≥50% myometrial invasion (MI). On the other hand, the GOG defines HIR based on a combination of age and the number of risk factors, including tumor grade2-3, lymphovascular space invasion (LVSI), or involvement of the outer third of the MI. For patients aged at least 70 years, one risk factor is required. For patients aged at least 50 years, two risk factors are required, and for patients under 50 years of age, all three risk factors are needed. In early ovarian cancer, adjuvant chemotherapy after successful tumor cell reduction surgery has not been shown to improve survival outcomes; However, in advanced ovarian cancer, a first-line chemotherapy regimen is typically recommended, which often consists of a combination of platinum-based drugs (such as cisplatin or carboplatin) and paclitaxel (Taxol). This combination therapy is widely recognized and used in clinical practice to enhance treatment efficacy (16).

## 9 Survival and prognosis

### 9.1 Survival outcome

The survival outcomes of SEOC are favorable, with reported 5-year survival rates ranging from 83% to 85.9% and 10-year survival rates ranging from 80.3% to 96% (13, 60). In comparison, stage III endometrial cancer has a 5-year overall survival rate of approximately 57% to 66% (42), while stage II endometrioid ovarian cancer has a survival rate of around 82% (61). SEOC confined to the ovaries and uterine corpus has a favorable prognosis, which may be associated with the prevalence of early-stage, low-grade, and endometrioid histology tumors in both locations (62, 63). In a study by Matsuo, K. et al. (63) a retrospective analysis compared the survival rates between stage I endometrial cancer and stage I SEOC with tumors in both locations being of endometrioid histology. The study found that the survival outcomes were similar between the two groups. Some studies suggest that SEOC patients with tumors in both locations being of endometrioid histology have better survival rates compared to patients with non-endometrioid histology (14, 25, 41). On the contrary, there are also studies that indicate no significant difference in survival rates between patients with endometrioid and non-endometrioid histology (64).

### 9.2 Prognosis factors

#### 9.2.1 Age and menopausal state

Age is an independent prognostic factor for SEOC patients (52, 64). A study in Italy analyzing 46 SEOC patients found that age affects patient prognosis, the 5-year survival rate for patients under 50 years old was 94.1%, while for those over 50 years old, it was 53.7% (64). Compared to premenopausal SEOC patients, postmenopausal patients have an increased risk of recurrence (52).

#### 9.2.2 CA125

CA125 is widely used in the postoperative follow-up and monitoring of ovarian cancer. An elevated CA125 level has also been shown to be a predictive factor for poor prognosis in endometrial cancer patients (65). There are also studies reporting the impact of preoperative CA-125 levels on prognosis. In a multicenter retrospective study conducted in South Korea in 2014, patients with normal CA-125 levels had significantly better progression-free survival (PFS) and overall survival (OS) compared to patients with elevated CA-125 levels (11). The diagnostic value of preoperative CA125 levels in SEOC requires further research to confirm.

#### 9.2.3 Lymphovascular space invasion

Lymphovascular space invasion (LVSI) refers to the presence of tumor cells within the capillary lumens of the lymphatic or microvascular drainage system of the primary tumor. A retrospective study from India analyzed 43 SEOC patients using a COX regression model in multivariate analysis and found that the presence of LVSI in both sites of the tumor is an independent prognostic factor for survival (41). In endometrial cancer patients, LVSI is an independent prognostic factor for lymphatic metastasis and distant recurrence, indicating an adverse survival outcome (66). LVSI is also an independent predictor of progression and survival in early-stage primary epithelial ovarian cancer patients (67).

#### 9.2.4 Tumor histological grade

Most studies have confirmed that SEOC is more common in early stage and low-grade tumors. Whether it’s endometrial cancer or ovarian cancer, a lower degree of tumor differentiation is typically associated with a worse prognosis. Research indicates a significant correlation between the histological grade of endometrial cancer and recurrence in SEOC patients (52). SEOC patients with high-grade lesions in both sites have a higher recurrence rate and significantly worse prognosis compared to those with low-grade lesions (13, 41, 64).

#### 9.2.5 Tumer stage

Some studies suggest that the staging of ovarian cancer in SEOC is a factor influencing its recurrence and prognosis (5, 11, 41, 52). In the study by Song, T. al (11)., they analyzed the 5-year progression-free survival (PFS) and overall survival (OS) of 123 SEOC patients. They found that staging significantly influenced the prognosis of ovarian cancer (PFS P = 0.019, OS P = 0.003), but it did not have a significant impact on endometrial cancer (PFS P = 0.534, OS P = 0.651). This suggests that patients with ovarian cancer in stages II-IV have a higher risk of recurrence and poorer prognosis.

Bese et al. (52) analyzed and compared 13 patients with recurrent SEOC and 18 patients without recurrent SEOC, and found that omental metastases were present in 10 patients (77%) in the recurrent group, indicating that omental metastases were significantly correlated with recurrent SEOC.

A study from Japan in 2019 found that single-factor and multi-factor analyses showed that cervical stromal invasion had a significant impact on PFS (Progression-Free Survival) and OS (Overall Survival) (58). Based on this research, it was suggested that prognostic factors for SEOC (double cancer) patients might be different from those of endometrial or ovarian cancer patients. However, further research is needed to validate this finding and gain a better understanding of the survival prognosis and related factors for SEOC.

There is controversy regarding whether lymph node metastasis affects the prognosis of SEOC patients. Some studies have shown that lymph node metastasis does not significantly affect the survival rate of SEOC patients (52). In the study by Turashvili et al. (68) a multifactorial analysis demonstrated an association between lymph node involvement (hazard ratio (HR) = 2.38, 95% CI 1.13-5.02, p = 0.023) and worse progression-free survival (PFS). This requires further research to better understand the impact of lymph node involvement on SEOC patients.

#### 9.2.6 Residual lesion

Recurrence of SEOC was significantly correlated with residual lesions after surgery. In the Bese, T. study, 8 out of 31 patients with SEOC had residual tumors, and 7 of them had recurrence (52). If the initial surgery did not include staging or achieve satisfactory debulking, the necessity of a second surgery should be considered. It is also advisable to consider more aggressive adjuvant therapy to improve survival outcomes and prognosis. The size of residual lesions after the initial surgery is an independent prognostic factor in ovarian cancer, with smaller residual lesions associated with a better prognosis. However, there have been no similar studies in SEOC, and further research is needed to confirm this in clinical practice.

#### 9.2.7 TP53 mutation

A multicenter retrospective study from the Netherlands in 2020 analyzed the molecular characteristics of SEOC patients and compared them with TCGA profiles. They found that SEOC patients had an enrichment of PTEN and CTNNB1 mutations and fewer TP53 mutations compared to cases with metastatic tumors. TP53 mutations are considered an independent predictor of poor prognosis. It is recommended to assess the TP53 mutation status in these patients using methods such as NGS (Next-Generation Sequencing) or immunohistochemistry. This can help stratify the risk in these patients for the consideration of systemic adjuvant therapy (31).

## 10 Conclusion

SEOC is clinically rare, characterized by early stages, low grade, and favorable prognosis. Accurate diagnosis and differentiation are of great significance for its management, treatment, and prognosis. Due to the difficulty of preoperative and intraoperative clinical diagnosis, reliance on postoperative pathological examination is necessary for diagnosis and differentiation, posing a significant challenge for clinical physicians in devising personalized diagnostic and treatment plans. With the continuous development of new technologies like genetic sequencing, there is hope for improved diagnostic accuracy, which in turn can aid in enhancing patient prognosis.

## Author contributions

WG: Writing – original draft, Funding acquisition. CB: Conceptualization, Writing – review & editing.
